# S1P_2_-Gα_12_ Signaling Controls Astrocytic Glutamate Uptake and Mitochondrial Oxygen Consumption

**DOI:** 10.1523/ENEURO.0040-21.2021

**Published:** 2021-07-13

**Authors:** Deepa Jonnalagadda, Yasuyuki Kihara, Richard Rivera, Jerold Chun

**Affiliations:** Translational Neuroscience Initiative, Sanford Burnham Prebys Medical Discovery Institute, La Jolla, CA 92037

**Keywords:** ponesimod, sphingolipids, lysophospholipids, fingolimod, siponimod, ozanimod

## Abstract

Glutamate is the principal excitatory neurotransmitter in the human brain. Following neurotransmission, astrocytes remove excess extracellular glutamate to prevent neurotoxicity. Glutamate neurotoxicity has been reported in multiple neurologic diseases including multiple sclerosis (MS), representing a shared neurodegenerative mechanism. A potential modulator of glutamate neurotoxicity is the bioactive lysophospholipid sphingosine 1-phosphate (S1P) that signals through five cognate G-protein-coupled receptors, S1P_1_–S1P_5_; however, a clear link between glutamate homeostasis and S1P signaling has not been established. Here, S1P receptor knock-out mice, primary astrocyte cultures, and receptor-selective chemical tools were used to examine the effects of S1P on glutamate uptake. S1P inhibited astrocytic glutamate uptake in a dose-dependent manner and increased mitochondrial oxygen consumption, primarily through S1P_2_. Primary cultures of wild-type mouse astrocytes expressed S1P_1,2,3_ transcripts, and selective deletion of S1P_1_ and/or S1P_3_ in cerebral cortical astrocytes, did not alter S1P-mediated, dose-dependent inhibition of glutamate uptake. Pharmacological antagonists, S1P_2_-null astrocytes, and Gα_12_ hemizygous-null astrocytes indicated that S1P_2_-Gα_12_-Rho/ROCK signaling was primarily responsible for the S1P-dependent inhibition of glutamate uptake. In addition, S1P exposure increased mitochondrial oxygen consumption rates (OCRs) in wild-type astrocytes and reduced OCRs in S1P_2_-null astrocytes, implicating receptor selective metabolic consequences of S1P-mediated glutamate uptake inhibition. Astrocytic S1P-S1P_2_ signaling increased extracellular glutamate, which could contribute to neurotoxicity. This effect was not observed with the FDA-approved S1P receptor modulators, siponimod and fingolimod. Development and use of S1P_2_-selective antagonists may provide a new approach to reduce glutamate neurotoxicity in neurologic diseases.

## Visual Abstract

**Figure:**
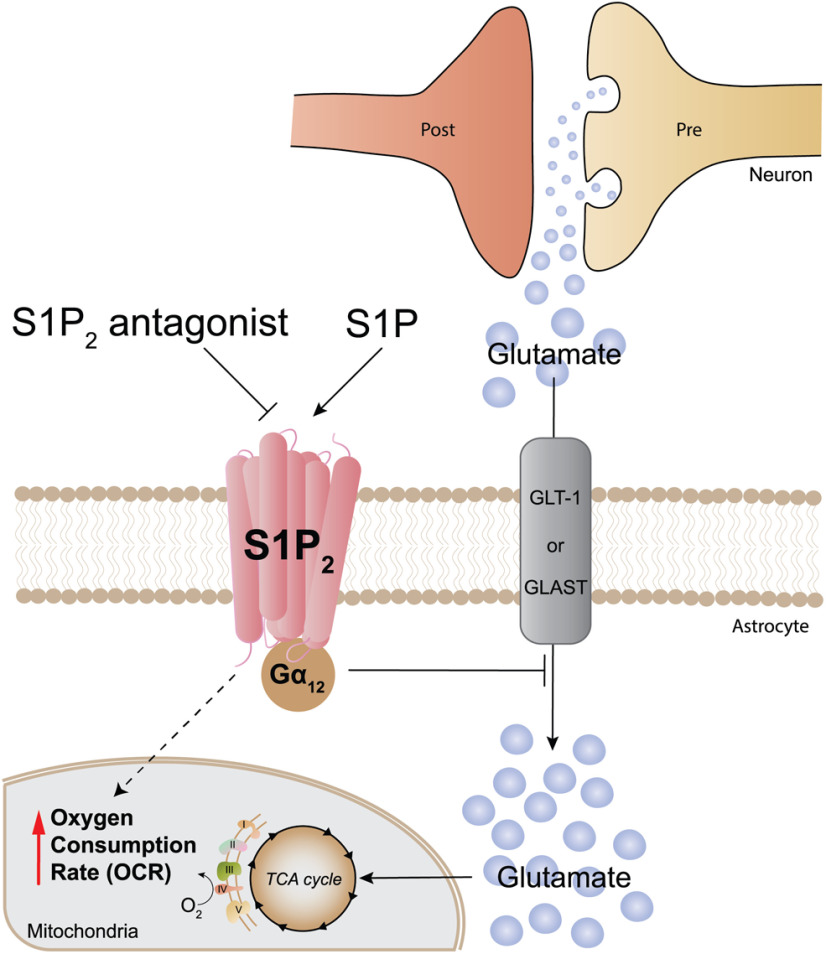

## Significance Statement

Extracellular glutamate is excitotoxic and its levels are controlled by astrocyte uptake. Sphingosine 1-phosphate (S1P) is a bioactive lipid originating from cell membrane sphingolipids and associates with carrier molecules like albumin, ApoM, and ApoA4 to produce cellular effects. S1P signals extracellularly through five G-protein-coupled receptors and it is found in higher concentrations in neurologic diseases like multiple sclerosis (MS) where excitotoxic neurodegeneration has been implicated. Here, we show that astrocytic S1P_2_ activation by S1P results in glutamate uptake inhibition to promote excitotoxic damage. S1P receptor modulators, including approved drugs for treating MS, e.g., fingolimod (FTY720) and siponimod (BAF312), do not engage S1P_2_, thus avoiding glutamate uptake inhibition. S1P_2_ antagonists may provide a means to reduce S1P-induced glutamate neurotoxicity and ameliorate neurologic diseases.

## Introduction

L-glutamate is the most abundant free amino acid in the brain, and it is the primary excitatory neurotransmitter (Lipton and Rosenberg, 1994; Vandenberg and Ryan, 2013; Lewerenz and Maher, 2015). Glutamate dysregulation has been associated with neurologic conditions such as epilepsy (Mathern et al., 1999; Barker-Haliski and White, 2015), Alzheimer’s disease (Masliah et al., 1996; Li et al., 1997; Scott et al., 2011), Huntington’s disease (André et al., 2010), amyotrophic lateral sclerosis (Bruijn et al., 1997; Ilieva et al., 2009), and multiple sclerosis (MS; Srinivasan et al., 2005; Baranzini et al., 2010; Muhlert et al., 2014; MacMillan et al., 2016; Macrez et al., 2016). The involvement of glutamate dysregulation in these neurologic diseases makes pharmacological modulation of glutamate uptake an attractive therapeutic target (Anderson and Swanson, 2000).

Astrocytes are the predominant cell type involved in the maintenance of glutamate homeostasis, which can be altered in disease states (Mahmoud et al., 2019). Astrocytes take up excess glutamate via the transmembrane expression of glutamate transporter-1 (GLT-1; Pines et al., 1992) and L-glutamate/L-aspartate transporter (GLAST; Storck et al., 1992) and then glutamate is converted to non-toxic glutamine (Henn et al., 1974; Su et al., 2003). Glutamate uptake is an ATP-driven process (McKenna, 2013), where the glutamate taken up by astrocytes enters the TCA cycle (McKenna et al., 1996) and aids in mitochondrial respiration (Sonnewald et al., 1993; Hertz and Hertz, 2003). Activated astrocytes have also been implicated in several disease states (Sofroniew, 2014; Groves et al., 2018; Ponath et al., 2018; Yamanaka and Komine, 2018; Siracusa et al., 2019), and in a recent report using a mouse model of MS, astrocytes were predominantly activated during the disease development phase and were suppressed by fingolimod (FTY720) treatment (*ieAstrocytes*; Groves et al., 2018). FTY720 is a prodrug approved as a medicine for MS patients that must be phosphorylated to become an active agent (FTY720-P), where it acts a structural analog of sphingosine 1-phosphate (S1P; Blaho and Hla, 2014) to target S1P_1,3,4,5_ (Brinkmann et al., 2002; Mandala et al., 2002). Additional S1P receptor modulators have recently become available for MS treatment that do not require phosphorylation, including the S1P_1,5_-selective modulators, ozanimod and siponimod (BAF312; Chun et al., 2021). As additional S1P receptor modulators are developed for central nervous system disease treatment, an understanding of how S1P signaling functions within the brain, including the receptors mediating these functions, becomes increasingly important. In the present study, primary astrocytes from wild-type and S1P receptor-null mouse cerebral cortices were combined with pharmacological assessments employing S1P receptor modulators to identify S1P receptor-mediated effects on glutamate uptake and mitochondrial respiration.

## Materials and Methods

### Mice

All animal procedures were conducted in accordance with Institutional Care and Use Committee guidelines of Sanford Burnham Prebys Medical Discovery Institute. Postnatal day (P)0–1 C57BL/6J pups were collected for primary astrocyte cultures. S1P_1_ astrocyte conditional (GFAP Cre) null As-S1P_1_^−/−^ mice, S1P_3_ null mice, As-S1P_1_/S1P_3_ double-null mice, and Gα_12_ heterozygous-null mice were on a C57BL/6J background. S1P_2_^−/−^ mice were on a BALB/c background. All the experiments were performed regardless of sex of the mouse.

### Primary astrocyte culture

Cerebral hemispheres were harvested from P0–P1 C57BL/6J pups and the meninges were removed and placed in DMEM/F12 (Life Technologies) containing penicillin and streptomycin (PS; Life Technologies), 50 μg/ml DNase I (Sigma-Aldrich), and 0.05% trypsin (Sigma-Aldrich). After gentle dissociation, the tissue was incubated for 30 min at 37°C. Trypsin was inactivated with DMEM/F12 containing 10% FBS (Gemini), and the cell suspension was centrifuged at 800 rpm for 5 min, resuspended in DMEM/F12 containing 10% FBS, and filtered through a 40 μm cell strainer (BD Biosciences). The cells were cultured in DMEM/F12 containing 10% FBS and passaged once. On their second passage, the cells were distributed into 24-well plates. After 24 h, the cells were serum-starved overnight, and experiments were conducted unless otherwise noted. FTY720-P-containing medium was replenished every 24 h over 3.5 d of exposure to ensure functional antagonism activity.

### Reagents

L-[3,4-^3^H]-glutamic acid was obtained from PerkinElmer. S1P and VPC23019 were obtained from Avanti Polar Lipids. FTY720-phosphate was obtained from Novartis Pharma, AG and Cayman Chemical. RP001, Y27632, and JTE013 were from Tocris Bioscience. BAF312 (Siponimod) was obtained from Novartis Pharma AG and Apexbio. Pertussis toxin (PTX) from *Bordetella pertussis* was obtained from List Biological Laboratories.

### Glutamate uptake assay

Serum-starved primary astrocytes (∼10^5^ cells per sample) were incubated with 2 μM L-sodium glutamate (Sigma) and 20 nM ^3^H-Glutamic acid. Cells were immediately stimulated with agonist or treated with vehicle for 1 h at 37°C. The cell supernatant was then aspirated, the primary astrocytes were washed thoroughly with cold HBSS containing Ca2^+^/Mg2^+^ ions, and then incubated with 0.25 mL of 0.2 N NaOH for 2 h at room temperature. EcoLume scintillation cocktail (MP Biomedicals) was added to the samples and radioactivity [counts per minute (cpm)] was measured with a scintillation counter (Beckman). The percentage (%) of glutamate uptake by astrocytes was calculated as the (cpm in the test sample × 100) / (cpm in the vehicle sample).

### Gene expression analysis

Gene expression was measured using TaqMan fast advance master mix and TaqMan probes for β-actin and S1P_1-5_ (ThermoFisher Scientific) and analyses were conducted on a Bio-Rad CFX 384 Touch Real-Time PCR Detection System. All reactions were performed in triplicate and the average Ct was used to calculate the receptor expression relative to β-actin.

### Seahorse analysis

An Agilent Seahorse XF Cell Mito Stress Kit was used to measure oxygen consumption rate (OCR). Briefly, primary astrocytes (7 × 10^4^/well) were plated on the Seahorse 24-well culture plate. After allowing the cells to acclimate in a 37°C non-CO_2_ incubator, the analyzer was calibrated, and the OCR was measured through sequential injection of electron transport chain inhibitors: (1) 1 μM oligomycin (ATP synthase inhibitor); (2) 1 μM FCCP (uncoupling agent); and (3) a combination of 0.5 μM rotenone (complex I inhibitor) and 0.5 μM antimycin A (complex III inhibitor). The non-mitochondrial respiratory rate (NMRR) corresponds to the OCR measurement after injection of rotenone and antimycin A. Basal OCR was calculated by subtracting NMRR from the initial recorded OCR (endogenous OCR). Proton leak was calculated by subtracting the NMRR from the OCR following oligomycin injection. Maximal OCR was calculated by subtracting the NMRR from the OCR following FCCP injection. Spare respiratory capacity is the difference between the maximal and the basal OCR.

### Statistical analysis

Statistics were analyzed using GraphPad Prism 8. All dose response curves are an average of two to three independent experiments with two technical replicates each. The data were fitted by performing nonlinear regression. All data are represented as mean ± SEM unless otherwise noted. A summary of the statistical analyses is presented in Table 1.

**Table 1.** Statistical table

Figure	Method performed
1*A*,*C*,*D*	Non-linear regression
1*E*	Two-way ANOVA/Sidak’s multiple comparisons test
2*A–C*	Non-linear regression
3*A–D*	Two-way ANOVA/Sidak’s multiple comparisons test
4*A*,*B*	Non-linear regression
5*C*	Unpaired *t* test
5*D–H*	Ordinary one-way ANOVA/Tukey’s multiple comparisons test
5*I*	Two-way ANOVA/Tukey’s multiple comparisons test

## Results

### S1P-mediated inhibition of glutamate uptake by astrocytes is dose-dependent and distinct from S1P receptor modulators

**Figure 1.**
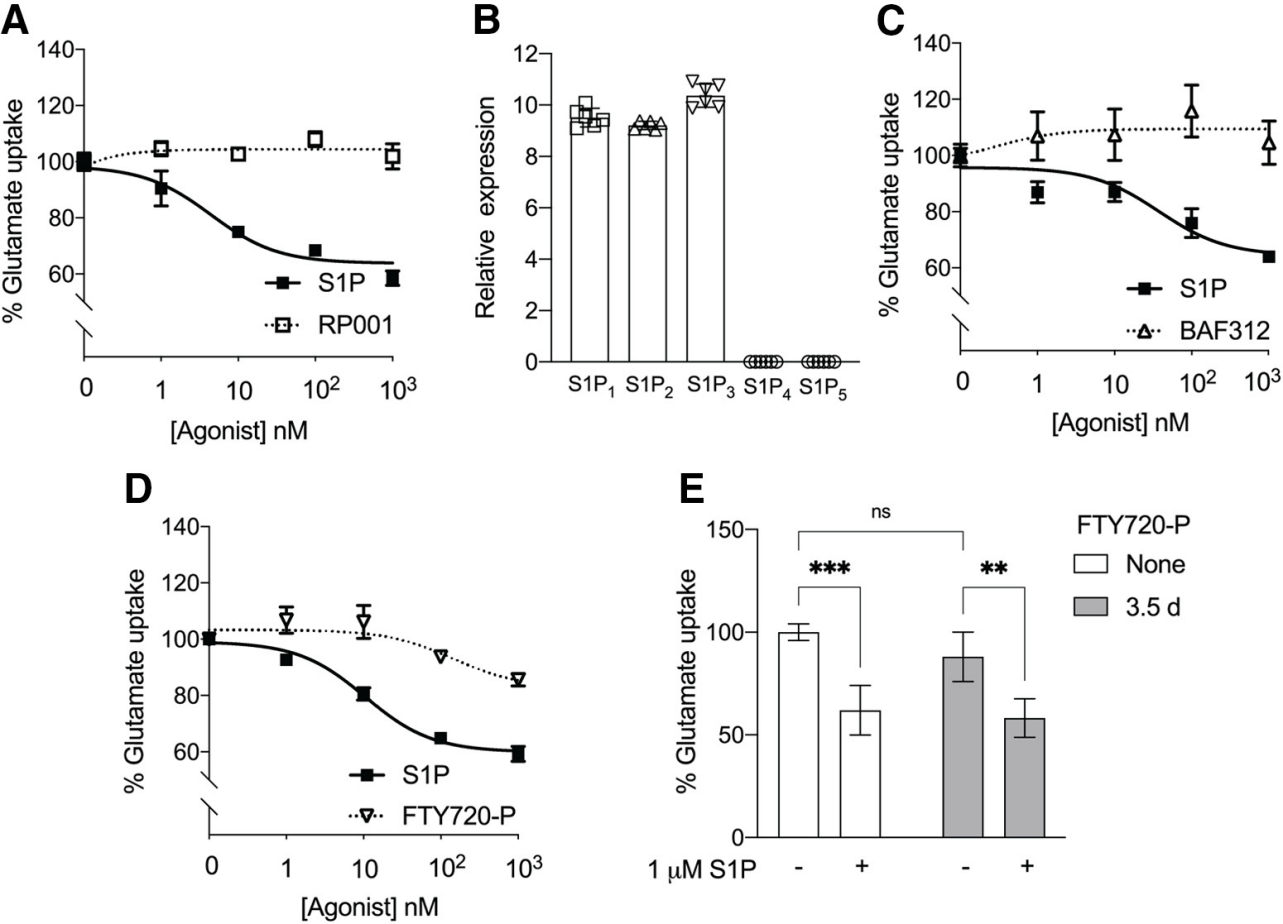
Inhibition of glutamate uptake by S1P contrasts with S1P receptor modulator effects. Primary astrocytes were incubated with labeled glutamate, S1P, or respective agonists for 1 h at 37°C. The glutamate uptake assay was performed as described. ***A***, S1P inhibited glutamate uptake by astrocytes in a dose-dependent manner, while the S1P_1_-specific agonist, RP001, did not. ***B***, The relative expression of S1P_1-5_ (normalized to β-actin) in wild-type primary astrocytes derived from cerebral cortices of P0 pups. ***C***, The S1P_1,5_ modulator, BAF312, did not induce glutamate uptake inhibition at the indicated doses. ***D***, The S1P_1,3,4,5_ modulator, FTY720-P, induced partial inhibition. ***A***, ***C***, ***D***, Data are from two independent experiments and curve fitting was performed by nonlinear regression. ***E***, Incubation with FTY720-P for 3.5 d, followed by stimulation with S1P, induced glutamate uptake inhibition similar to no FTY720-P pretreatment controls. Data are representative of three independent experiments. Error bars indicate (mean ± SD). Statistics are calculated by two-way ANOVA performing Sidak’s multiple comparisons test (***p < 0.0005, **p < 0.005; ns, not significant).

To understand how S1P affects astrocyte glutamate uptake, mouse primary astrocytes were cultured in the presence of radiolabeled glutamate and treated with increasing doses of S1P for 1 h. The percent of astrocytic glutamate uptake decreased with an increasing dose of S1P, resulting in a ∼40% reduction in glutamate uptake at the highest doses (Fig. 1*A*). An S1P_1_-selective agonist, RP001, did not inhibit glutamate uptake (Fig. 1*A*), indicating that S1P_1_ is not the primary receptor responsible for inhibiting glutamate uptake. Quantitative RT-PCR revealed that mouse primary astrocytes express mRNAs for S1P_1,2,3_ but not S1P_4,5_ (Fig. 1*B*). To determine whether S1P receptor modulators with distinct receptor subtype engagement affect astrocyte glutamate uptake, BAF312 and FTY720-P were tested on primary astrocytes. The S1P_1,5_ modulator, BAF312 (Chun et al., 2021), did not alter glutamate uptake at any dose tested under agonist conditions (Fig. 1*C*). FTY720-P, a high-affinity S1P receptor modulator for S1P_1,3,4,5_, did produce a 10% reduction of glutamate uptake at 1 μM, the highest concentration examined (Fig. 1*D*). A 3.5 d pretreatment with FTY720-P (functional antagonist conditions) did not produce an observable change in S1P-mediated astrocyte glutamate uptake inhibition compared with non-treated controls (Fig. 1*E*). These data show that astrocyte-mediated control of extracellular glutamate occurs through an S1P dose-dependent mechanism and is likely mediated through S1P_2_.

### Receptor-null astrocytes show that glutamate uptake inhibition is not mediated through S1P_1_ or S1P_3_

**Figure 2.**
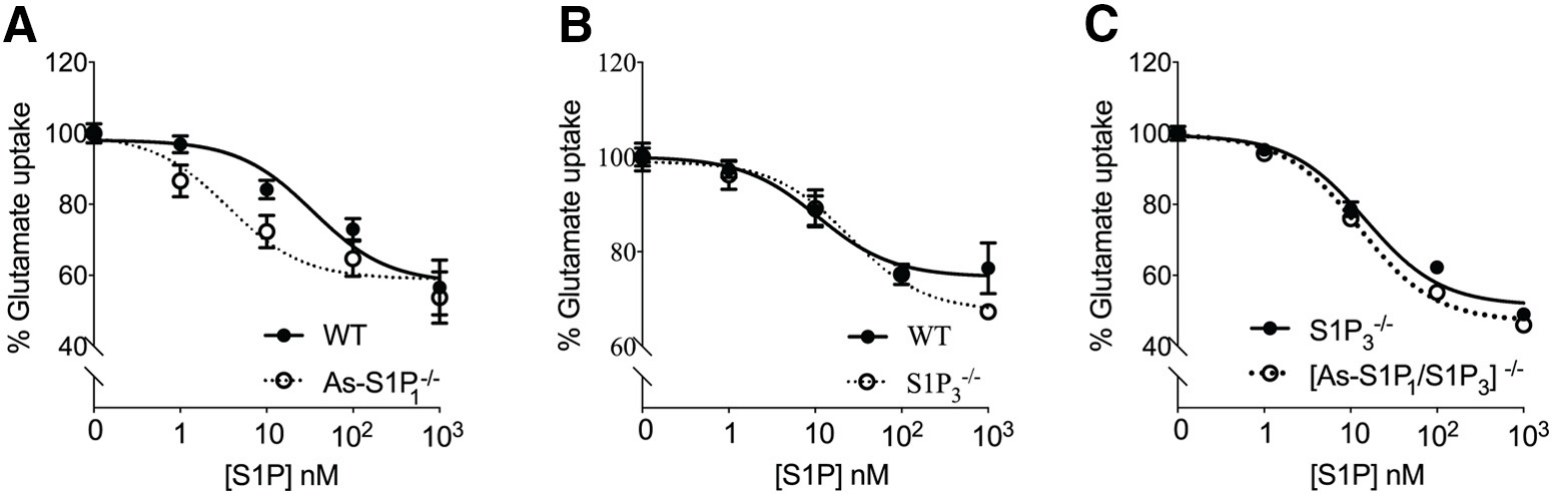
Glutamate uptake inhibition is not eliminated in S1P_3_-null and astrocyte-specific (As)-S1P_1_/S1P_3_-double-null astrocytes. Primary astrocytes from As-S1P_1_, S1P_3_, and As-S1P_1_/S1P_3_ receptor-null mice were incubated with labeled glutamate and S1P for 1 h at 37°C. ***A***, Glutamate uptake in As-S1P_1_^−/−^ astrocytes exhibited a slight left shift (decrease in glutamate uptake) compared with littermate controls. S1P_3_^−/−^ (***B***) and As-S1P_1_/S1P_3_^−/−^ (***C***) astrocytes exhibited control levels of glutamate uptake. Data in each panel are from three independent experiments. Curve fitting was performed by nonlinear regression.

To understand how S1P_1_ and/or S1P_3_ influence astrocyte glutamate uptake, primary astrocytes derived from mice lacking these receptors were incubated with increasing doses of S1P (Fig. 2). A moderate decrease (left shift of curve) in glutamate uptake was observed in the absence of S1P_1_ on astrocytes compared with wild-type astrocytes (Fig. 2*A*), indicating that S1P_1_ might aid in glutamate uptake homeostasis. On the other hand, in the absence of S1P_3,_ glutamate uptake was inhibited in a manner similar to that of wild-type astrocytes (Fig. 2*B*). To confirm the role of S1P_1_ on astrocyte glutamate uptake, As-S1P_1_-null and S1P_3_-null mice were crossed to obtain As-S1P_1_/S1P_3_-null mice. Primary astrocytes cultured from As-S1P_1_/S1P_3_-null mice exhibited a similar trend to that of S1P_3_-null with increasing doses of S1P, indicating that neither S1P_1_ nor S1P_3_ are primarily involved in astrocyte glutamate uptake (Fig. 2*C*).

### S1P_2_ mediates S1P-induced astrocyte-mediated glutamate uptake inhibition

**Figure 3.**
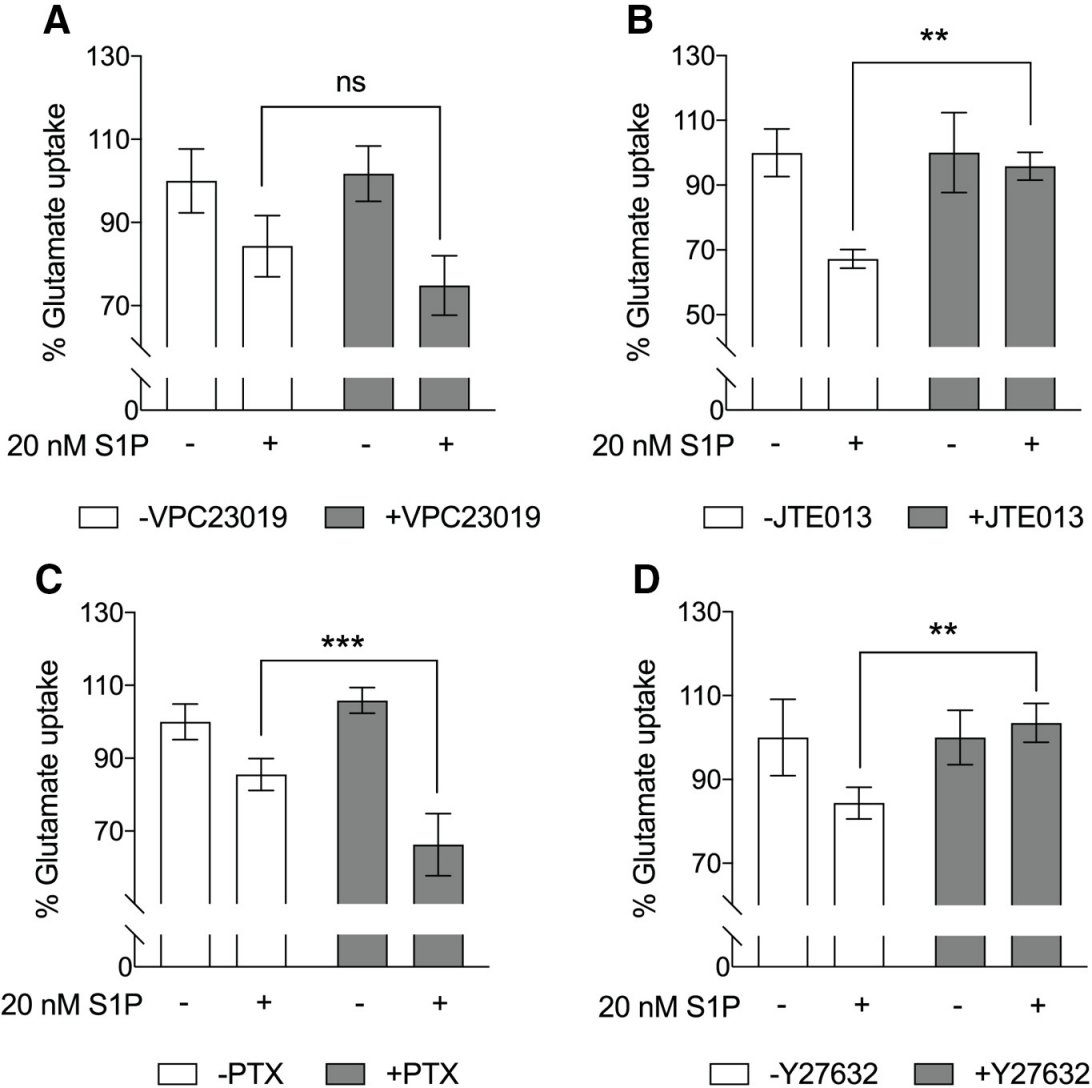
Pharmacological inhibition of S1P_2_, but not S1P_1_ or S1P_3_, eliminates astrocytic inhibition of glutamate uptake by S1P. Primary astrocytes were preincubated (***A***) with or without 10 μM VPC23019, an antagonist for S1P_1_ and S1P_3_, for 45 min, (***B***) 10 μM JTE 013, an S1P_2_ antagonist, for 30 min, (***C***) 200 ng/mL PTX overnight, and (***D***) 10 μM Y27632 for 30 min at 37°C followed by incubation with or without 20 nM S1P for 1 h. Glutamate uptake assays were performed as described. Data in each panel are representative of two independent experiments (*n* = 3 or 4). Error bars represent mean ± SD; ns, not significant; **p < 0.005, ***p < 0.001, two-way ANOVA with Sidak’s multiple comparisons test.

Pharmacological antagonists were employed to test whether S1P_2_ plays a role in astrocyte glutamate uptake inhibition. Primary astrocytes were preincubated with VPC23019, an antagonist for S1P_1_ and S1P_3_, and then stimulated with S1P for 1 h (Fig. 3*A*). No significant differences in glutamate uptake were observed compared with non-treated controls. Combined with prior results (Figs. 1, 2), these data indicated that S1P_2_ is primarily involved in inhibiting glutamate uptake. Indeed, the S1P_2_ antagonist, JTE013, rescued the inhibitory effect of S1P on astrocyte glutamate uptake (Fig. 3*B*). S1P_2_ couples with G_i_, G_12/13_, or G_q_ proteins to initiate downstream signaling pathways. Therefore, astrocytes were pretreated with PTX (an inhibitor of the G_i_ proteins by ADP-ribosylation) and Y27632 (an inhibitor of Rho kinase downstream of G_12/13_) to deermine the pathways involved in S1P_2_-mediated glutamate uptake inhibition. Pretreatment with PTX did not alter S1P-induced glutamate uptake inhibition (Fig. 3*C*). Conversely, Y27632 completely rescued the S1P-induced inhibition of astrocyte glutamate uptake (Fig. 3*D*). These results indicated that Gα_12/13_ signaling downstream of S1P_2_ inhibits glutamate uptake in astrocytes.

**Figure 4.**
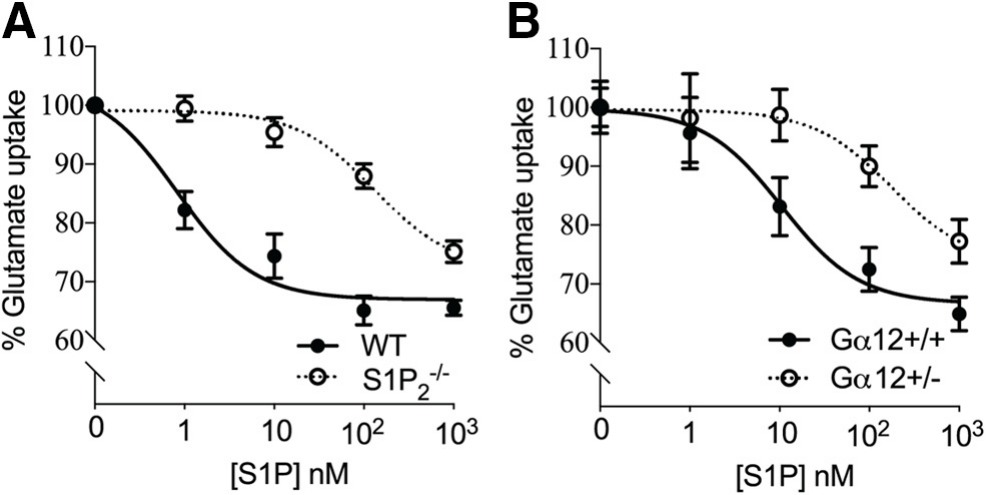
Genetic removal of S1P_2_ or Gα_12_ downstream signaling reduces astrocytic glutamate uptake inhibition by S1P. Primary astrocytes from S1P_2_-null or Gα_12_-hemizygous-null mice were incubated with labeled glutamate and S1P for 1 h at 37°C. Glutamate uptake in S1P_2_^−/−^ (***A***) and Gα_12_^+/−^ (***B***) astrocytes exhibited a right shift (increase in glutamate uptake) when compared with controls. Data in each panel are from three independent experiments. Curve fitting was performed by nonlinear regression.

In addition to pharmacological studies, glutamate uptake in astrocytes genetically-null for S1P_2_ or hemizygously null for Gα_12_ was tested. Astrocytes null for S1P_2_ (Fig. 4*A*) or hemizygously null for Gα_12_ (Fig. 4*B*) exhibited an increase (right shift of curve) in glutamate uptake when compared with controls which supports S1P_2_ and Gα_12_ playing a role in inhibiting glutamate uptake. The possible involvement of additional S1P receptors or non-receptor mechanisms (e.g., non-cell autonomous effects) in S1P-induced glutamate uptake inhibition could not be excluded since glutamate uptake was not completely restored by genetic ablation of S1P_2_ or reduction of Gα_12_.

### S1P affects mitochondrial respiration through S1P_2_

**Figure 5.**
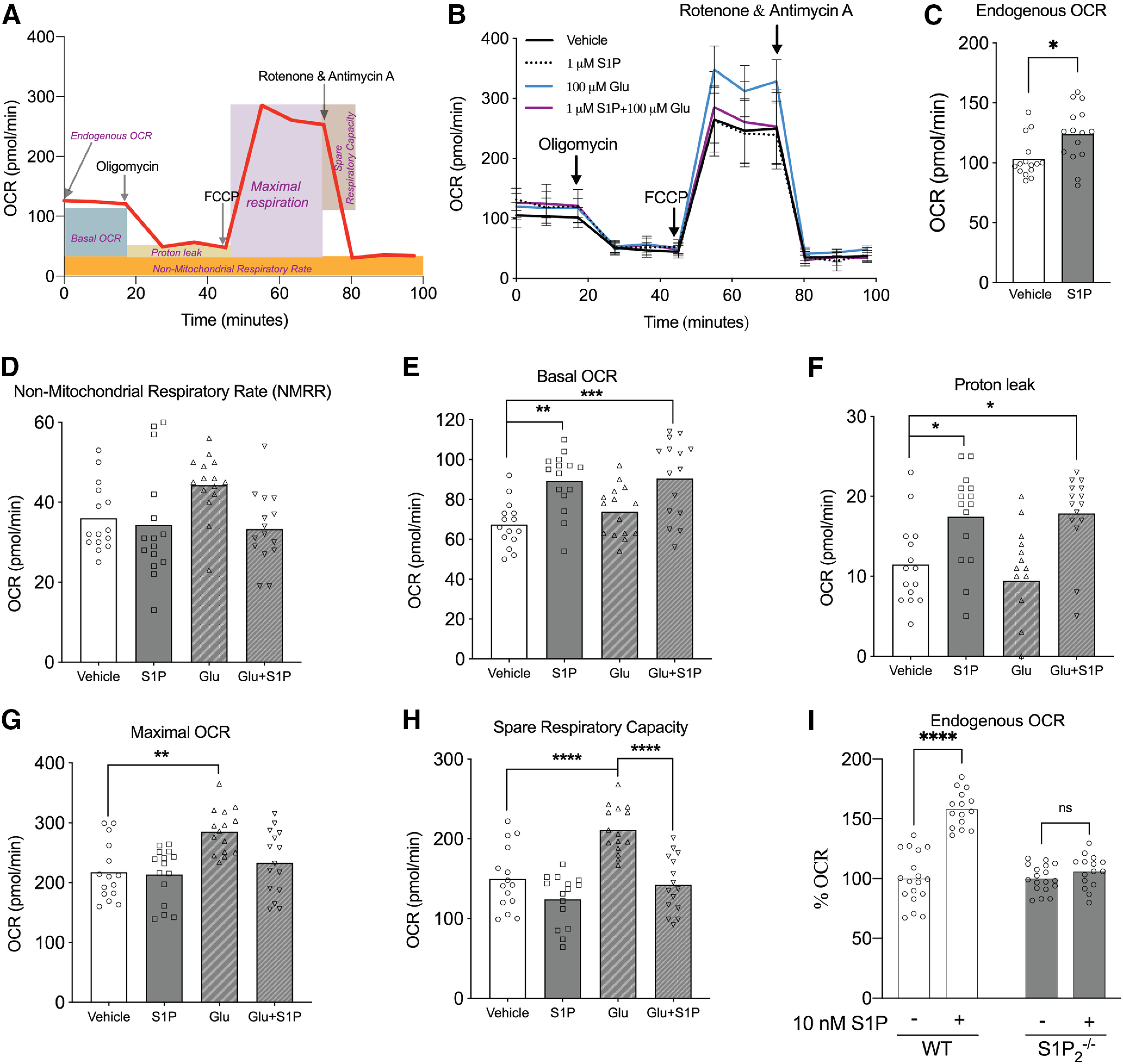
S1P increases astrocyte mitochondrial oxygen consumption rate (OCR) that is S1P_2_ dependent. ***A***, Schematic of Seahorse Mito Stress analysis. ***B***, Primary astrocytes were exposed to four conditions: vehicle, 100 μM glutamate, 1 μM S1P, or a combination of glutamate and S1P for 1 h at 37°C. After washing, Seahorse assay medium containing 1 mM pyruvate and 10 mM glucose was added and the OCR measured with an Agilent Seahorse XF24 Cell Mito Stress kit. OCR was measured three times per injection and was read in triplicate. ***C***, S1P increased endogenous OCR when compared with vehicle *p < 0.05 unpaired *t* test. Non-mitochondrial respiratory rate (NMMR = OCR after rotenone/antimycin A addition; ***D***), basal OCR (= endogenous OCR – NMRR; ***E***), maximal OCR (= OCR after FCCP addition – NMRR; ***F***), proton leak (= OCR after oligomycin injection – NMRR; ***G***), and spare respiratory capacity (= maximal OCR – basal OCR; ***H***) is represented under different conditions as indicated, *n* = 5 with OCR measured in triplicate at each step. Data are representative of two independent experiments with similar results. ***D–H***, Ordinary one-way ANOVA with Tukey’s multiple comparisons test (*p < 0.05, **p < 0.005, ***p ≤ 0.0005, ****p < 0.0001). ***I***, Wild-type and S1P_2_ null primary astrocytes were incubated with Seahorse assay medium containing 1 mM pyruvate and 10 mM glucose followed by treatment with 10 nM S1P for 1 h. OCR was then measured using the Agilent Seahorse XF24 analyzer. % OCR in the presence of S1P was calculated with respect to vehicle as indicated, ****p < 0.0001, two-way ANOVA with Tukey’s multiple comparisons test. ns, not significant.

Astrocytic glutamate uptake is an ATP-driven process known to affect mitochondrial function and health (Sonnewald et al., 1993; Hertz and Hertz, 2003). To understand how exposure to glutamate and S1P affects mitochondrial health, Seahorse analyses were used to measure the rate of oxygen consumption (Fig. 5*A*). Wild-type primary astrocytes were exposed to vehicle, glutamate, S1P, or a combination of glutamate and S1P for 1 h, then washed and incubated with Seahorse assay media containing glucose and pyruvate. The OCR was measured through the sequential addition of oligomycin, FCCP, and rotenone/antimycin A (Fig. 5*B*). S1P increased the endogenous OCR when compared with vehicle (Fig. 5*C*), indicating an increased energy demand. In all employed conditions, no significant difference in the NMRR was observed (Fig. 5*D*). Glutamate did not change basal OCR (Fig. 5*E*) or proton leak (Fig. 5*F*), whereas S1P increased basal OCR and proton leak regardless of the presence or absence of glutamate. These data suggest that S1P signaling directly increases mitochondrial OCR, which may be independent from intracellular glutamate pools (Hamilton and Attwell, 2010). However, the maximal OCR (Fig. 5*G*) and the spare respiratory capacity (Fig. 5*H*) were significantly elevated by glutamate exposure, but were not affected by S1P, indicating that a glutamate pool is crucial for responding to increased energy demand. The observed elevation in spare respiratory capacity on glutamate exposure was diminished by the addition of S1P (Fig. 5*H*), indicating that S1P inhibition of glutamate uptake might be responsible for this decrease.

Lastly, to test whether the endogenous astrocyte OCR is altered in an S1P_2_-dependent manner, astrocytes derived from wild-type and S1P_2_-null mutant mice were exposed to S1P for 1 h, and the OCR was measured. Exposure of wild-type astrocytes to S1P produced a statistically significant increase in OCR that was not observed in S1P_2_ null astrocytes (Fig. 5*I*), indicating that S1P-S1P_2_ signaling increases mitochondrial respiration.

## Discussion

This is the first study to report that astrocyte S1P-S1P_2_ signaling affects glutamate uptake. S1P concentration gradients are perturbed in neurologic diseases (Cartier and Hla, 2019) and could contribute to glutamate neurotoxicity by disrupting glutamate homeostasis to produce increased extracellular glutamate (Mahmoud et al., 2019). Glutamate levels are controlled in part by astrocytes that express glutamate transporters capable of removing excess glutamate following neurotransmission (Anderson and Swanson, 2000; Mahmoud et al., 2019). S1P was found to inhibit glutamate uptake in a dose-dependent manner that was primarily because of the activation of S1P_2_ through a Gα_12_ pathway. Glutamate also affects astrocyte mitochondrial metabolism via entry into the TCA cycle (Jackson and Robinson, 2018), which was reported to be important for the maintenance of surrounding neurons in a stroke model (Fiebig et al., 2019). Seahorse analyses indicated that inhibition of glutamate uptake through S1P_2_ may affect mitochondrial health, implicating a change in the metabolic state of the astrocytes that can be abrogated by the loss of S1P_2_.

The results from the present study were obtained using primary cultures of astrocytes from newborn S1P_2_-null mice on a BALB/c background. Previous research reported seizure activity in S1P_2_-null mice which was attributed to background strain differences and possible glutamate excitotoxicity during a developmentally discrete period of young adulthood (between P25 and P45; MacLennan et al., 2001; Ishii et al., 2002; Akahoshi et al., 2011). S1P_2_ is also required for auditory and vestibular function wherein its constitutive loss results in early postnatal cochlear and vestibular degeneration (MacLennan et al., 2006; Herr et al., 2007; Kono et al., 2007). This involvement of S1P-S1P_2_ is consistent with the rare occurrence of autosomal recessive hearing impairment from S1P_2_ missense variants (Santos-Cortez et al., 2016). This further underscores links between S1P-S1P_2_, where modification by developmental age and genetic background could result in additional pathology beyond glutamate neurotoxicity.

Hypothetically, S1P receptor modulators could also increase extracellular glutamate levels because they engage S1P receptors. However, none of the assayed S1P receptor modulators inhibited glutamate uptake, which is consistent with their avoidance of S1P_2_ engagement. RP001 (S1P_1_-selective), BAF312 (siponimod, Mayzent; S1P_1/5_ selective; Jackson et al., 2011; Gergely et al., 2012; Al-Salama, 2019; Fig. 1*C*), and phosphorylated FTY720 (fingolimod/Gilenya; S1P_1,3,4,5_ selective; Brinkmann et al., 2002; Mandala et al., 2002) did not substantively inhibit glutamate uptake at 10 nM, with FTY720-P showing only a 10% reduction in glutamate uptake at 1 μM, which may reflect partial engagement of S1P_2_ at this high concentration (Sobel et al., 2015). These results are consistent with the lack of high-affinity S1P_2_ engagement by these modulators as compared with S1P for both agonist and functional antagonist properties. These results support the development of S1P_2_ antagonists to restore astrocytic glutamate uptake, which adds to the list of widely diverse activities of S1P_2_ (Adada et al., 2013), including the known effects on CCL5 expression that reduce neuroinflammation (Yester et al., 2015). The prevention of glutamate uptake inhibition by potential S1P_2_ antagonists currently under development could be beneficial in preventing glutamate neurotoxicity for a range of neurologic diseases with associated high brain levels of S1P. In summary, S1P-S1P_2_-Gα_12_-Rho/ROCK signaling controls astrocytic glutamate uptake and mitochondrial oxygen consumption, representing another important role for S1P signaling in central nervous system health and disease.

## Synthesis

Reviewing Editor: Karen Szumlinski, University of California at Santa Barbara Decisions are customarily a result of the Reviewing Editor and the peer reviewers coming together and discussing their recommendations until a consensus is reached. When revisions are invited, a fact-based synthesis statement explaining their decision and outlining what is needed to prepare a revision will be listed below. The following reviewer(s) agreed to reveal their identity: Anthony Reeder, Tomomi Furihata.

Upon consultation with two expert reviewers, we have reached the decision of “Revise and Re-review” as some concerns were raised by both reviewers, but the amount of work required to revise the study should take less than 2 months to complete. Both reviewers considered the data to be potentially impactful. Reviewer 1 has provided a list of relatively minor revisions and would like to see the implications of the findings expanded. Reviewer 2 was more critical of the report, particularly with respect to its descriptive nature. That being said, Reviewer 2 acknowledges that some important new leads are generated by the report and they provide a number of suggestions for improvement. Please find the reviewers’ comments below. I look forward to receiving your revised report. Given the pandemic, should you require additional time to complete your revision, please simply notify the eNeuro team.

Reviewer 1

Demonstrates that S1P2 is important in astrocyte uptake of toxic glutamate.

Is it possible that S1P2 blockade would inhibit learning and memory, especially if dose were too high?

Mention potential benefit in neurodegenerative CNS diseases at end of Discussion.

Grammar changes will enhance impact and clarity.

Abstract:

By combining... This key sentence of abstract is awkward, passive tense, and does not agree in verb usage.

S1P1,2 & 3. Modulation

Taken together... Awkward: two clauses, and the receptor targets of FTY and SIP should be mentioned.

Page 2, top

S1P is a bioactive “cell membrane” lipid

is found in higher concentrations

SIP increases glutamate uptake inhibition

Key word S1P2 astrocyte are most important facets

Introduction:

Page 3, middle

including the S1P1 & 5-selective

Methods:

Page 5

Glutamate uptake assay. Serum-starved

Bottom

Is this antimycin A?

Briefly describe the role of each of these inhibitors.

Results:

Page 8

(Fig. 2). A moderate left shift in glutamate uptake, i.e., less uptake in k/o

3B). Since S1P2 couples with Gi, G12/13, or Gq proteins, pertussis toxin (PTX

Bottom

3B). S1P2 couples with Gi, G12/13, or Gq proteins. Therefore, pertussis toxin (PTX

did not block S1P-induced GUI (Fig. 3C).

page 9

(Fig. 4B) exhibited a right shift, i.e., more uptake in k/o

To understand how S1P-dependent

Delete: using a Seahorse XF24 Analyzer per the manufacturer’s instructions

Page 10

S1P2-dependent manner

Discussion

intermediate35 and astrocytic

rewrite: Previous research reported S1P2...

page 11

tenses do not agree: of S1P-S1P2 relevant to glutamate homeostasis, which could counter

Figures:

Fine The WT vs. experimental conditions are consistently labeled.

Reviewer 2

The authors have identified involvement of S1P2 receptor in astrocyte glutamate homeostasis through regulation of the glutamate uptake activity. The paper may contain new findings that contribute to the field of astrocyte physiology and neurochemistry. However, in my impression, the study is incomplete, and thus is difficult to consider for the publication as it is.

1. First, it is unclear why the authors focused on mitochondrial functions in this study. No clear explanation is provided. Then, while S1P2 could be involved in regulation of mitochondrial functions, the data directly linking S1P2-mediated GUI to the functional alteration of mitochondria are missing. Two evens guided by S1P2 signaling might be independent. It is also difficult to understand how the partial glutamate uptake inhibition leads to complete inhibition of glutamate-mediated OCR increase.

2. Why is the treatment period of the antagonist so long? The 3.5-day exposure seems unusual.

3. The uptake activity is better to be normalized by mg protein, because the cell number affects the cpm value unless the cell density is confluent.

4. The writing organization is premature, especially in the discussion section. In addition, several errors can be found: e.g., the last sentence of the introduction - “GUI” is inappropriate; in the method section - concentration of antinomycin A is missing.

5. In the title, what does “differentially reduced by S1P receptor modulators” mean? GUI is rescued only by JTE013 in Fig. 3, but it is not surprising given Fig 1 and Fig 2.
